# Clinical and molecular genetic risk determinants in adult long QT syndrome type 1 and 2 patients: Koponen et al. Follow-up of adult LQTS patients

**DOI:** 10.1186/s12881-018-0574-0

**Published:** 2018-04-05

**Authors:** Mikael Koponen, Aki S. Havulinna, Annukka Marjamaa, Annukka M. Tuiskula, Veikko Salomaa, Päivi J. Laitinen-Forsblom, Kirsi Piippo, Lauri Toivonen, Kimmo Kontula, Matti Viitasalo, Heikki Swan

**Affiliations:** 1 Heart and Lung Center, Helsinki University Central Hospital, Helsinki, Finland; 2 Department of Health, National Institute for Health and Welfare, Helsinki, Finland; 3 Department of Medicine, Helsinki University Central Hospital and University of Helsinki, Helsinki, Finland; 4 synlab Humane Genetik Munich, Munich, Germany; 5 Department of Genetics, United Medix Laboratories Ltd, Helsinki, Finland

**Keywords:** Long QT syndrome, Cardiac arrhythmia, Risk stratification, β-blocker, Implantable cardioverter-defibrillator

## Abstract

**Background:**

Long QT syndrome (LQTS) is an inherited cardiac disorder predisposing to sudden cardiac death (SCD). We studied factors affecting the clinical course of genetically confirmed patients, in particular those not receiving β-blocker treatment. In addition, an attempt was made to associate risk of events to specific types of *KCNQ1* and *KCNH2* mutations.

**Methods:**

A follow-up study covering a mean of 18.6 ± 6.1 years was conducted in 867 genetically confirmed LQT1 and LQT2 patients and 654 non-carrier relatives aged 18–40 years. Cox regression models were used to evaluate the contribution of clinical and genetic risk factors to cardiac events.

**Results:**

In mutation carriers, risk factors for cardiac events before initiation of β-blocker included LQT2 genotype (hazard ratio [HR] = 2.1, *p* = 0.002), female gender (HR = 3.2, *p* < 0.001), a cardiac event before the age of 18 years (HR = 5.9, p < 0.001), and QTc ≥500 ms (vs < 470 ms, HR = 2.7, *p* = 0.001). LQT1 patients carrying the *KCNQ1* D317N mutation were at higher risk (HR = 3.0–3.9, p < 0.001–0.03) compared to G589D, c.1129-2A > G and other *KCNQ1* mutation carriers after adjusting for gender, QTc duration, and cardiac events before age 18. *KCNH2* c.453delC, L552S and R176W mutations associated with lower risk (HR = 0.11–0.23, p < 0.001) than other *KCNH2* mutations.

**Conclusions:**

LQT2 (compared to LQT1), female gender, a cardiac event before age 18, and long QT interval increased the risk of cardiac events in LQTS patients aged 18 to 40 years. The nature of the underlying mutation may be associated with risk variation in both LQT1 and LQT2. The identification of high-risk and low-risk mutations may enhance risk stratification.

**Electronic supplementary material:**

The online version of this article (10.1186/s12881-018-0574-0) contains supplementary material, which is available to authorized users.

## Background

Long QT syndrome (LQTS) is a hereditary cardiac disorder characterized by impaired repolarization properties of cardiomyocytes which predispose to ventricular arrhythmias, syncope and sudden cardiac death (SCD). [1] A total of 16 genes associate with LQTS, and mutations in *KCNQ1* or *KCNH2* genes cause the most common subtypes LQT1 and LQT2, respectively. [2] According to recent ESC guidelines, β-blocker treatment should be initiated if QTc is prolonged, and it may be useful even with normal QTc. [3, 4] Implantable cardioverter-defibrillator (ICD) should be applied in more severe cases. [5, 6]

Presently, genotype and mutation location can be applied as a part of risk stratification. [7–11] As the number of molecularly tested mutation carriers increases, mutation-specific assessment might enable more individually tailored patient management strategies. [12] However, the data available for mutation-specific risk stratification are still limited. [12–16] Previous studies assessing the clinical course in genotyped subjects have included children [7, 8] or patients treated with β-blockers. [9–11, 17–19] In LQTS, the risk associated with gender and genotype is age-related, [10, 11, 17, 19] and β-blocker treatment impacts disparate subgroups of patients differently. [17–19] In the current study, the clinical course without β-blocker treatment was explored in genotyped LQT1 and LQT2 patients aged 18–40 years. In addition, we studied the association of six different LQTS-causing mutations with prognosis of the LQT1 and LQT2 patients.

## Methods

### Study population

The study population was drawn from the Finnish Inherited Arrhythmic Disorder Research Registry established in 1991 and comprising over 4000 molecularly tested subjects. The inclusion criteria were 1) genetically confirmed *KCNQ1* or *KCNH2* mutation, or genetically confirmed non-carrier status of the family-specific LQTS mutation, 2) and the age of more than 18 years at follow-up end. A questionnaire (Additional file 1) was sent to the study subjects and collected data included occurrence of syncope, setting in which syncope occurred, and data regarding β-blocker therapy. Compliance was defined as forgetting or not taking medication once a month or more often. The decision whether to initiate β-blocker therapy was made by the treating physician upon establishment of the diagnosis.

Data of all deaths during the follow-up were obtained from Statistics Finland by means of social security number search. ICD and pacemaker implantations, and left cardiac sympathetic denervations (LCSD) were identified using the Finnish Hospital Discharge Register (National Institute for Health and Welfare). Medical records were acquired for patients who had device therapy, underwent LCSD, suffered an aborted cardiac arrest (ACA), or died. Collected ICD data included implantation indications, complications, revisions, and ICD discharges. Autopsy documents of patients who died during the follow-up were evaluated. The study was approved by the Ethical Review Committee of Helsinki University Hospital, and a written informed consent was obtained from the study subjects. The Ministry of Social Affairs and Health consented for the participation of deceased subjects.

The follow-up study started from the age of 18 years and ended when the subject 1) returned the questionnaire, 2) turned 40 years [to avoid the effect of acquired cardiac disease] or 3) was deceased, which ever occurred first. The end point for statistical analyses was cardiac event comprising LQTS-related syncope, ACA, appropriate ICD shock, or SCD. LQTS-related syncope was defined as a transient loss of consciousness that was abrupt in onset and offset, and triggered by one of the following factors: swimming, other sports, loud noise, or startle, to avoid inclusion of vasovagal events. [9] Resuscitation events that required external defibrillation were defined as ACA. A death was regarded as being SCD if it was abrupt in onset without evident cause if witnessed, or was not explained by any other cause if it occurred in an unwitnessed setting such as sleep.

Direct DNA sequencing and restriction enzyme assays were used in identification of *KCNQ1* and *KCNH2* mutations as previously described. [20, 21] Mutations were categorized by mutation type as missense or non-missense (nonsense, frameshift, splice site, insertion or deletion) mutations, and by their location as described previously. [10, 11] Patients carrying more than one LQTS mutation (*n* = 7) were excluded from the comparison of the clinical characteristics and the multivariate risk analyses, but were included in the sections depicting device therapy. The specific single mutations included in the final study population are detailed in Additional file 2: Table S1. LQT1 Finnish founder (FF) mutations *KCNQ1* G589D and *KCNQ1* c.1129-2A > G, and LQT2 FF mutations *KCNH2* R176W and *KCNH2* L552S were combined to form the FF mutation population for LQT1 and LQT2, respectively. Non-carrier family members of the familial *KCNQ1* and *KCNH2* mutations served as the comparison group. All study subjects were of Finnish origin.

### Statistical analyses

Clinical characteristics were analyzed using chi-square and Fisher’s exact tests for categorical, and Wilcoxon rank-sum and Kruskal-Wallis one-way ANOVA tests for continuous variables. Kaplan-Meier methods were used to depict the cumulative incidence rate (=cumulative probability) of first cardiac event after the age of 18 years by genotype, gender, QTc interval, and mutation. The QTc cut-offs used were based on previous LQTS studies. [4, 7, 18] The significance of the differences was tested by the log-rank test. Multivariate Cox proportional hazards regression models were used to evaluate the independent contribution of genetic and clinical risk factors to first cardiac event after 18 years of age. Survival was also evaluated by assessing incidence rates of first cardiac events per person-years. All cumulative incidence graphs, log-rank tests, and primary Cox regression and incidence rate analyses were censored at the initiation of β-blocker medication. Secondary Cox regression and incidence rate analyses (Medical Treatment paragraph) were carried out including the follow-up time with time-dependent β-blocker medication. No violation of the proportional hazards assumption was detected as tested by log-log graphs. A separate QTc missing covariate was used for mutation carriers whose QTc data were unavailable (*n* = 35). No statistically significant interactions were discovered in interaction term analyses. All Cox regression models were adjusted for gender, QTc duration, cardiac events before the age of 18, and family membership using robust sandwich estimators. Statistical analyses were carried out using SPSS version 22. A 2-sided *p*-value ≤0.05 was interpreted as statistically significant.

## Results

A total of 2723 subjects fulfilled the inclusion criteria. The final study population (*n* = 1521) consisted of 14 subjects who died during the follow-up, 1495 subjects who responded (55%) to the inquiry, and additional 12 subjects with device therapy drawn from the Hospital Discharge Register. The study population comprised 867 LQTS mutation carriers (617 *KCNQ1*, 243 *KCNH2*, and seven with > 1 *KCNQ1* or *KCNH2* mutation), and 654 non-carrier relatives.

The final study cohort had 263 families, and 190 (22%) of the mutation carriers were probands. The total follow-up time without β-blocker medication in a subgroup of 1420 subjects was 18.6 ± 6.0 years. There were 285 subjects who had β-blocker medication at some point of the study, and the mean follow-up time with medication was 6.2 ± 5.4 years. Nonresponders (*n* = 2723–1521 = 1202) had a higher proportion of males than subjects of the final study population (52% vs 36%, *p* < 0.001). There was no difference in the proportion of LQTS subtypes, or mean QTc duration between these two groups.

### Clinical characteristics

The characteristics of the patients with a single mutation are shown in Table 1 and with more than one mutation in Additional file 2: Table S2**.** Characteristics of the non-carrier relatives are presented in Additional file 2: Table S3. Altogether seven of the ten deaths in mutation carriers, and none of the four deaths in non-carrier relatives were arrhythmia-related. Among mutation carriers seven (1%) suffered a SCD and eight (1%) at least one ACA. In LQT1 2% and in LQT2 3% of the cardiac events were fatal. Female mutation carriers were more often probands (26% vs 15%, *p* = 0.001), and had a longer QTc (473 vs 454 ms, *p* < 0.001) as compared with males. Altogether, QTc duration ≥500 ms was measured in 132 (15%) mutation carriers. One family had 7, three families had 3, and the remaining families had 0–2 cardiac events.

**Table 1:** Characteristics of the mutation carriers at the age of 18–40 years^a^

	All patients		LQT1				LQT2			
			Non-FF		FF		Non-FF		FF	
	LQT1	LQT2	D317N	Other	G589D	c.1129-2A > G	c.453delC	Other	L552S	R176W
N (%)	617 (72)	243 (28)	20 (3)	72 (12)	453 (73)	72 (12)	23 (10)	61 (25)	73 (30)	86 (35)
Female	396 (64)	157 (65)	17 (85)	48 (67)	282 (62)	49 (68)	11 (48)_a, b_^c^	33 (54)_a_	56 (77)_b_	57 (66)_a, b_
Age, y	36.3 ± 6.2	36.1 ± 6.8	36.5 ± 5.5	35.5 ± 7.2	36.3 ± 6.1	37.4 ± 5.7	35.1 ± 8.2	35.2 ± 7.1	37.0 ± 6.0	36.3 ± 6.9
QTc, ms	467 ± 40	465 ± 41	492±51_a_^c^	473 ± 43_b_	465 ± 40_b_	466 ± 28_b_	466 ± 39_a, b, c_	487 ± 45_a,_	466 ± 42_b_	448 ± 29_c_
Proband	112 (18)_1_^b^	78 (32)_2_	1 (5)	18 (25)	76 (17)	17 (24)	0_a_	34 (56)_b_	22 (30)_c_	22 (26)_c_
β-blocker	184 (30)	65 (27)	11 (55)_a_	30 (42)_a, b_	126 (28)_a, b_	17 (24)_b_	7 (30)_a, b_	31 (51)_a_	14 (19)_b_	13 (15)_b_
ICD	9 (2)_1_	11 (5)_2_	1 (5)	3 (4)	5 (1)	0	0_a, b_	9 (15)_a_	2 (3)_a, b_	0_b_
Pacemaker	5 (1)_1_	8 (3)_2_	0	0	3 (1)	2 (3)	0_a, b_	6 (10)_a_	0_b_	2 (2)_a, b_
LCSD	1 (0.2)	1 (0.4)	0	1 (1)	0	0	0	1 (2)	0	0
CE	69 (11)_1_	43 (18)_2_	8 (40)_a_	10 (14)_a, b_	44 (10)_b_	7 (10)_b_	1 (4)_a_	25 (41)_b_	9 (12)_a_	7 (8)_a_
Syncope^d^	62 (10)_1_	39 (16)_2_	7 (35)_a_	8 (11)_a, b_	40 (9)_b_	7 (10)_b_	1 (4)_a_	22 (36)_b_	9 (12)_a_	7 (8)_a_
ACA^e^	4 (1)	4 (2)	0	1 (1)	3 (1)	0	0	3 (5)	1 (1)	0
SCD^f^	4 (1)	3 (1)	0	1 (1)	3 (1)	0	0	3 (5)	0	0
CE without BB	55 (10)_a_	36 (16)_b_	5 (25)_a_	7 (10)_a, b_	36 (8)_b_	7 (10)_a, b_	1 (4)_a_	20 (33)_b_	8 (11)_a_	7 (8)_a_
CE with BB	16 (9)	9 (14)	3 (27)	3 (10)	10 (8)	0	0	7 (22)	2 (14)	0
CE age, y^g^	26.1 ± 5.9_1_	24.0 ± 5.7_2_	29.5 ± 5.7	25.6 ± 7.0	25.6 ± 5.7	25.5 ± 5.5	18.2 ± 0	23.8 ± 5.5	25.6 ± 7.0	23.6 ± 4.9
CE before age 18	74 (12)	30 (12)	6 (30)_a_	17 (24)_a_	46 (10)_b_	5 (7)_b_	1 (4)	15 (25)	6 (8)	8 (9)

Parameters shown as n (%), or mean ± SD

^a^Patients with > 1 LQTS-causing mutation (*n* = 7) are excluded

^b^Subscript numbers (1 or 2) indicate that the LQT1 and LQT2 patients have statistically significant difference (p < 0.05)

^c^Subscript letters (a, b, c or d) indicate that at least one group differs from the other three groups as tested separately within LQT1 and LQT2 patient groups. Groups with different subscript letters (a, b, c or d) have statistically significant difference after Bonferroni correction (*p* < 0.05)

^d^Triggered by swimming, sport, loud noise or startle

^e^A resuscitation that required external defibrillation

^f^Not explained by any other cause and abrupt in onset if witnessed

^g^The first cardiac event at the age of 18–40 years

*ACA*: aborted cardiac arrest, *BB*: β-blocker, *CE*: cardiac event, *FF*: Finnish founder, *ICD*: implantable cardioverter-defibrillator, *LCSD*: left cardiac sympathetic denervation, *SCD*: sudden cardiac death, *SD*: standard deviation

### Risk factors for cardiac events before β-blocker treatment

**Fig. 1:**
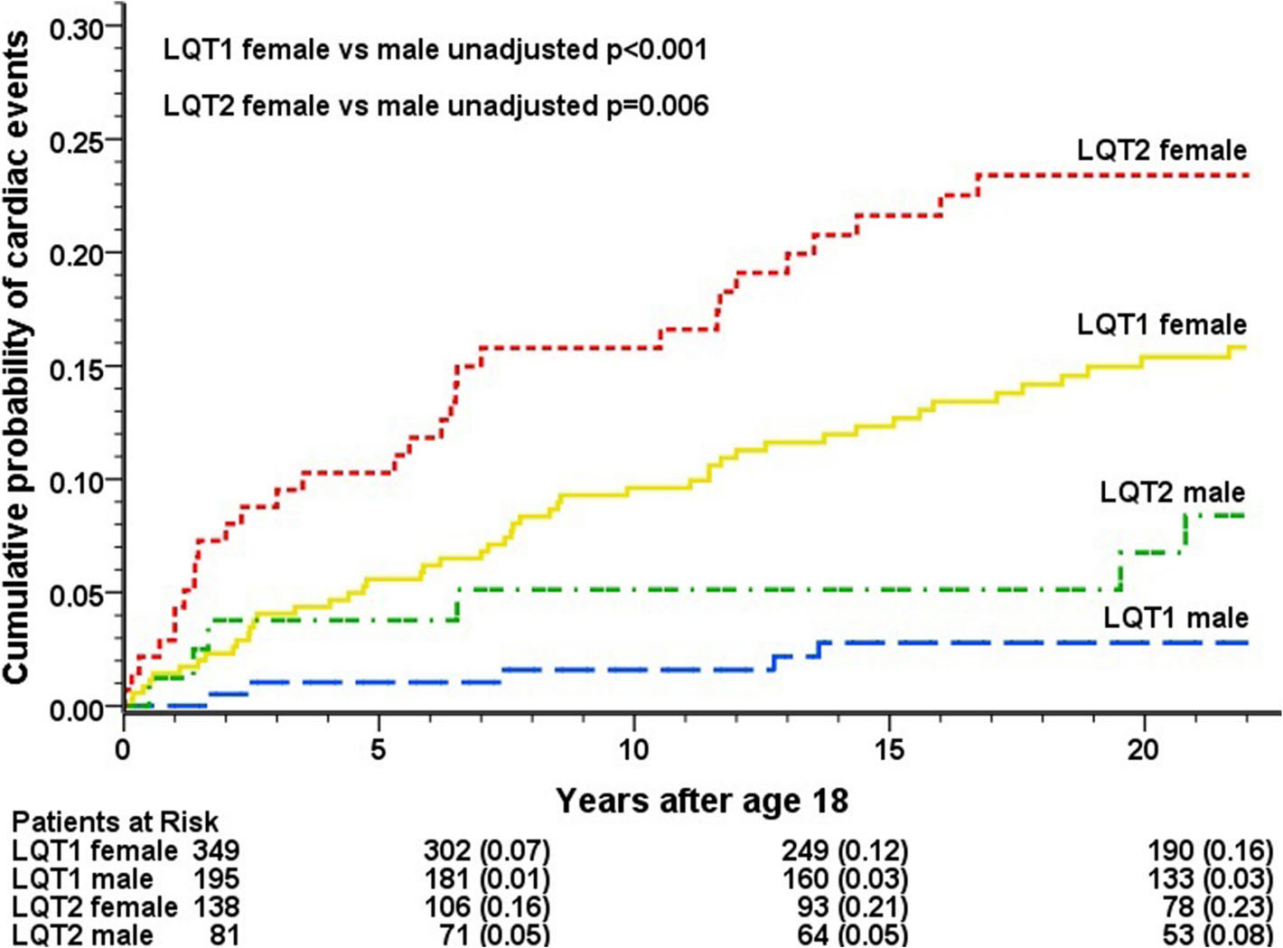
Cumulative incidence of cardiac events in LQT1 and LQT2 patients by gender before initiation of β-blocker treatment at the age of 18–40 years

LQT2 genotype was associated with a higher risk of cardiac events in comparison to LQT1 (cumulative probability 18% vs 11%, *p* = 0.01; HR = 2.1, *p* = 0.002, Table 2). Both LQT1 and LQT2 females were more often symptomatic than males (cumulative rate 16% vs 3%, *p* < 0.001, for LQT1; and 23% vs 8%, *p* = 0.01, for LQT2, Figure 1), with a hazard ratio of 3.2 for the female versus male comparison (p < 0.001). The risk was distinctly higher in patients who were symptomatic before the age of 18 years (cumulative rate 52% vs 9%, *p* < 0.001; HR = 5.93, p < 0.001). QTc duration ≥500 ms increased the risk 2.7-fold compared to QTc < 470 ms (*p* = 0.001). We repeated the analyses after excluding FF mutation carriers, and the results regarding genotype, gender, symptoms before age 18, and QTc duration were similar.

**Table 2:** Cox regression model: Adjusted risk of cardiac events at the age of 18–40 years in LQT1 and LQT2 patients before initiation of β-blocker medication^a^

	Hazard ratio	95% confidence interval	*P*-value
LQT2 vs LQT1	2.11	1.33–3.34	0.002
Female vs male	3.18	1.71–5.91	< 0.001
CE vs no CE before age 18	5.93	3.72–9.44	< 0.001
QTc ≥500 vs < 470 ms	2.66	1.53–4.64	0.001
QTc ≥500 vs 470–499 ms	2.22	1.23–4.00	0.01
QTc 470–499 vs < 470 ms	1.20	0.71–2.04	0.50

^a^Patients with > 1 LQTS-causing mutation (*n* = 7) are excluded

The model was adjusted for family membership using robust sandwich estimators

A separate QTc missing covariate was used for patients whose QTc data were unavailable (*n* = 35)

*CE*: cardiac event

The risk of cardiac events was higher in mutation carriers than in non-carrier relatives even after adjustment for QTc, gender, and cardiac events before age 18 (Table 3). In comparison with non-carrier relatives with QTc < 440 ms, mutation carriers with QTc < 440 and ≥ 440 ms had a 4.2-fold (*p* = 0.01) and an 11.1-fold (*p* < 0.001) risk, respectively, of suffering a cardiac event.

**Table 3:** Cox regression model: Adjusted risk of cardiac events at the age of 18–40 years in mutation carriers and non-carrier relatives before initiation of β-blocker medication^a^

	Hazard ratio	95% confidence interval	*P*-value
Non-carrier^b^	1 (reference)	–	–
*KCNQ1* G589D	6.06	3.49–10.5	< 0.001
*KCNQ1* c.1129-2A > G	6.83	3.19–14.6	< 0.001
*KCNQ1,* other mutations^c^	8.02	3.43–18.8	< 0.001
*KCNQ1* D317N	23.7	11.0–51.1	< 0.001
*KCNH2* c.453delC	3.86	1.91–7.79	< 0.001
*KCNH2* R176W	5.87	2.89–11.9	< 0.001
*KCNH2* L552S	7.80	3.86–15.8	< 0.001
*KCNH2,* other mutations^c^	33.3	18.4–60.3	< 0.001

^a^Patients with > 1 LQTS-causing mutation (n = 7) are excluded

^b^Mean QTc was significantly longer in all eight mutation carrier groups mentioned in the Table 3 than in non-carrier relatives (QTc 424 ± 26 and 410 ± 22 ms in female and male non-carriers, respectively). The cumulative probability of cardiac events was 2% in non-carrier relatives

^c^The *KCNQ1* and *KCNH2* mutations in the study are listed in Additional file 2: Table S1

The model was adjusted for gender, QTc duration, cardiac events before age 18, and family membership

**Fig. 2:**
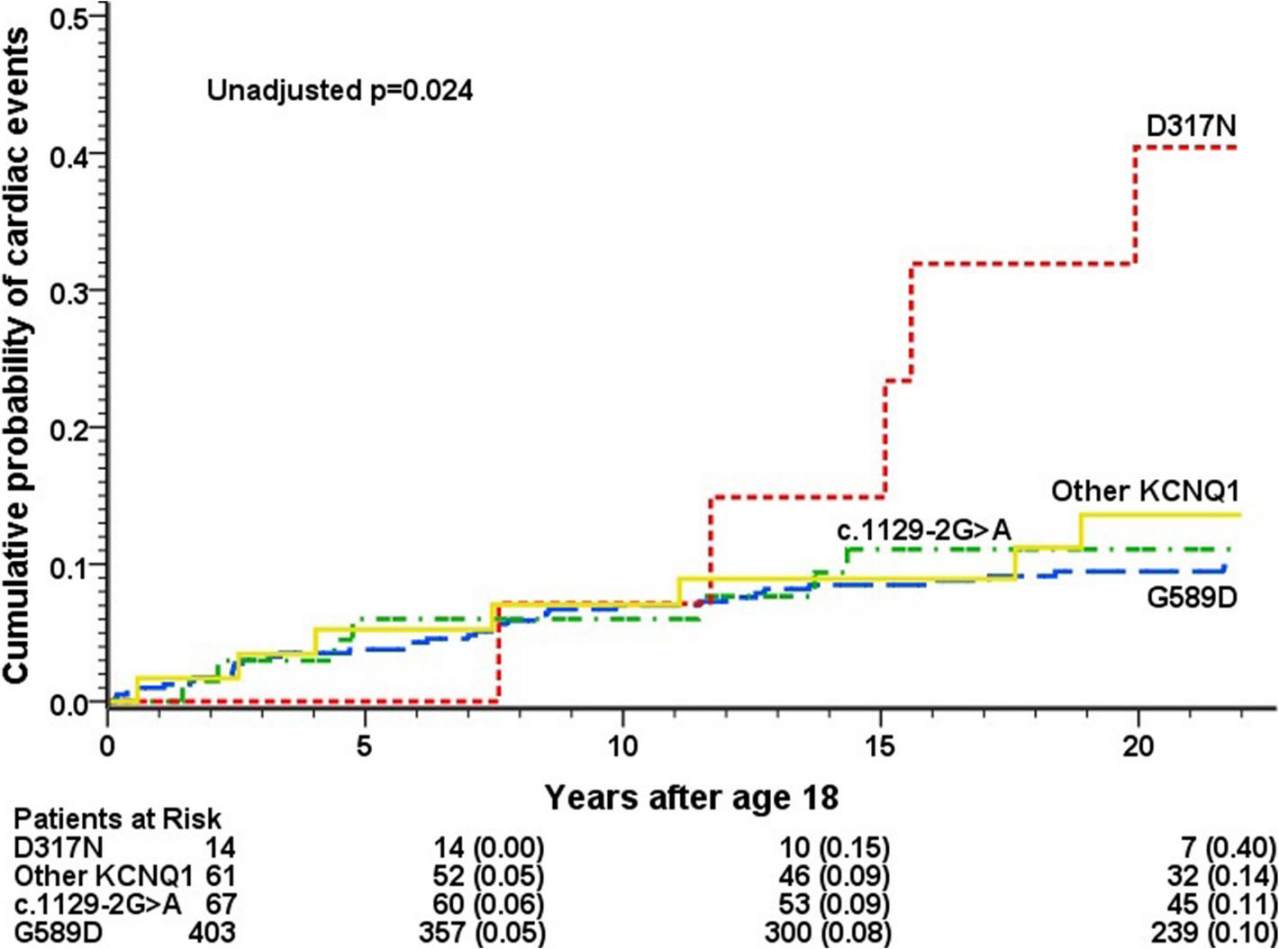
Cumulative incidence of cardiac events in LQT1 patients by mutation before initiation of β-blocker treatment at the age of 18–40 years

**Fig. 3:**
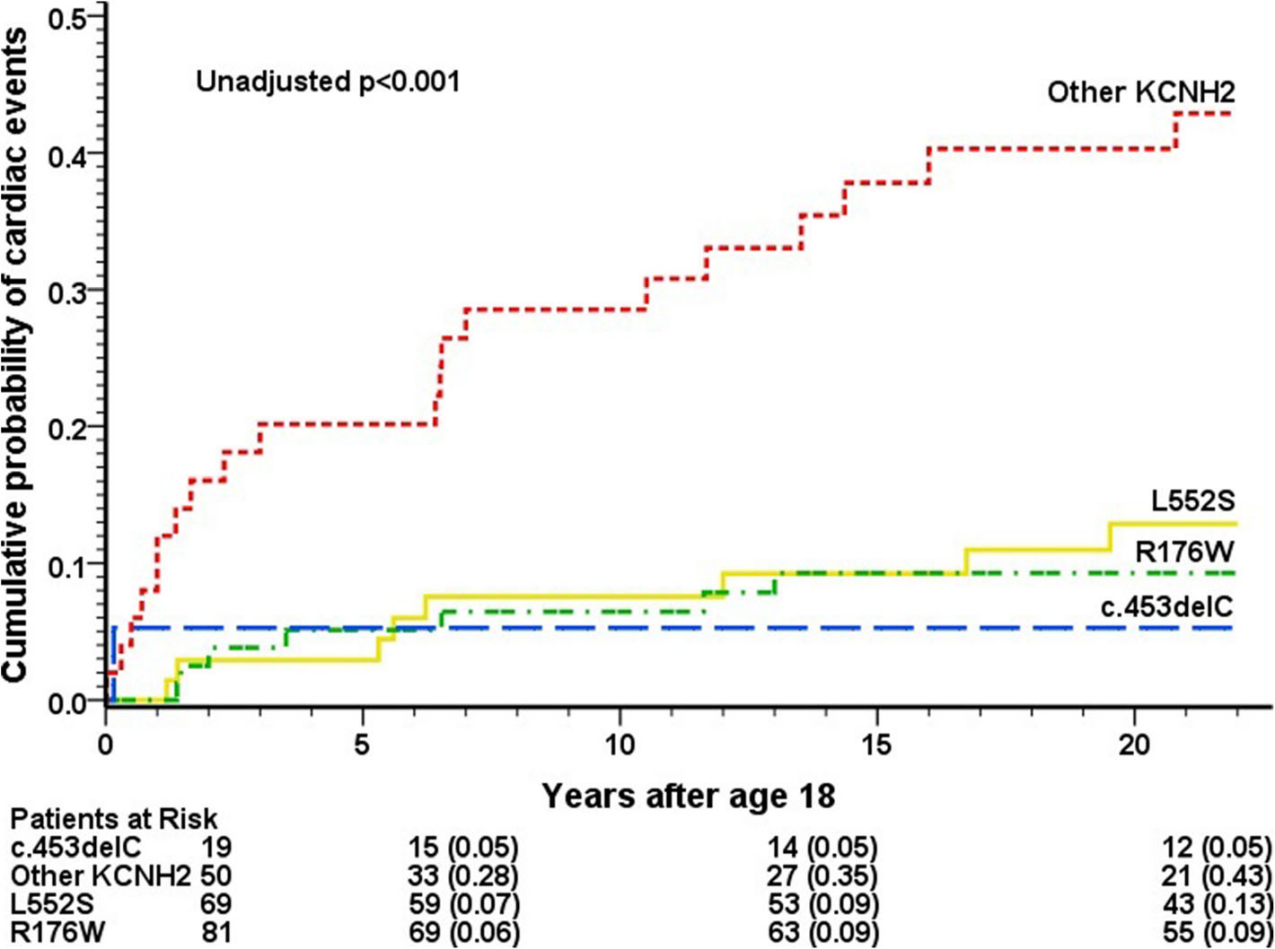
Cumulative incidence of cardiac events in LQT2 patients by mutation before initiation of β-blocker treatment at the age of 18–40 years

Upon pairwise comparison, *KCNQ1* D317N mutation carriers showed a higher risk of cardiac events than G589D, c.1129-2G > A or other *KCNQ1* mutation carriers (cumulative probability 40%, 10%, 11% and 14%, respectively, *p* = 0.002–0.047, Figure 2; HR = 3.0–3.9, *p* < 0.001–0.03). Risk or rate of events did not differ between G589D, c.1129-2G > A and other *KCNQ1* mutation carriers. Among LQT2 patients the carriers of the c.453delC, L552S or R176W mutation had fewer cardiac events than carriers of other *KCNH2* mutations (cumulative rate 5%, 13%, 9% and 43%, respectively, *p* < 0.001–0.013, Figure 3; HR = 0.11–0.23, p < 0.001).

An analysis of the effect of the mutation site (Table 1S) indicated that the cumulative rate of cardiac events was higher in patients carrying a missense *KCNQ1* non-FF mutation in pore-loop than in non-pore-loop region (37% vs 7%, p = 0.01). In a pairwise comparison, pore-loop mutation carriers also had a higher rate of events than carriers of a *KCNQ1* G589D or c.1129-2G > A mutation (*p* = 0.02–0.001), but there was no difference between non-pore-loop and FF mutation carriers (*p* = 0.48–0.53). A similar analysis of the LQT2 patients showed that among missense *KCNH2* non-FF mutation carriers there was a tendency of higher event rate in patients with a non-pore-loop than a pore-loop mutation (58% vs 28%, *p* = 0.14). Non-pore-loop mutation carriers had a higher event rate than *KCNH2* L552S or R176W mutation carriers (*p* < 0.001), and the event rate was similar between carriers of a pore-loop and a FF mutation (*p* = 0.054–0.15).

The rate of cardiac events was the same regardless of whether the mutation had been inherited from mother or father as tested separately among all *KCNQ1, KCNQ1* G589D, and all *KCNH2* mutation carriers (data were available for 459 *KCNQ1* and 150 *KCNH2* mutation carriers).

### β-blocker treatment

β-blocker medication was initiated to 249 mutation carriers at a mean age of 22.3 and 22.7 years in LQT1 and LQT2 patients, respectively. Use of medication was more common in non-FF than FF patients (45% vs 25%, *p* < 0.001), and in females than males (34% vs 20%, *p* < 0.001). Altogether 27 patients suffered a cardiac event during the medication, including SCD in four patients. Breakthrough events were more common in non-FF than FF mutation carriers (16% vs 7%, *p* = 0.04). In patients to whom β-blocker was prescribed, the medication was associated with 60–81% reduction in the risk of first cardiac event at the age of 18–40 years (p < 0.001, Table 4).

**Table 4:** Time-dependent Cox regression model: Adjusted risk factors for cardiac events at the age of 18–40 years in the 249 LQT1 and LQT2 patients who were treated with β-blocker medication^a^

	Hazard ratio	95% confidence interval	*P*-value
BB vs no BB in non-FF	0.40	0.29–0.57	< 0.001
BB vs no BB in *KCNQ1* G589D	0.19	0.09–0.41	< 0.001
BB vs no BB in other FF	0.30	0.18–0.51	< 0.001
Non-compliance vs compliance	1.87	1.35–2.59	< 0.001
Side effects vs no side effects	1.08	0.80–1.47	0.61

^a^Patients with > 1 LQTS-causing mutation (*n* = 7) are excluded

β-blocker treatment was considered in a time-dependent manner

The effect of β-blocker treatment is shown separately for carriers of non-FF, *KCNQ1* G589D, or other FF mutation

The β-blockers used were bisoprolol (43%), propranolol (33%), atenolol (12%), metoprolol (10%), acebutolol (2%), and betaxolol (1%)

The model was adjusted for gender, QTc duration, cardiac events before age 18, and family membership

A separate QTc missing covariate was used for patients whose QTc data were unavailable

BB = β-blocker

Non-compliance to β-blocker medication associated with a 1.9-fold (*p* < 0.001) increase in the risk of cardiac events. However, the incidence rates of cardiac events in non-compliant patients were 52.0 and 34.0 per 1000 person-years before and after initiation of the medication, respectively, indicating a protective impact also in these patients.

### Concomitant medications

Treatment with psychotropic agents was equally common in mutation carriers and non-carrier relatives (antidepressants: 5% vs 5%, *p* = 1.00; antipsychotics: 2% vs 1%, *p* = 1.00; anxiolytics: 0.4% vs 0.6%, *p* = 1.00, respectively). However, mutation carriers who had suffered a cardiac event reported more often taking daily antidepressant drug at the end of the follow-up (12% vs 3%, *p* = 0.02).

### Device therapy and LCSD

Characteristics of the 39 patients with ICD, pacemaker or LCSD are detailed in Additional file 2: Table S4. The incidence rate of cardiac events showed reduction after ICD implantation: 152.4 and 56.8 per 1000 person-years before and after implantation, respectively. Similarly, cardiac events decreased after pacemaker implantation: 82.3 and 0 per 1000 person-years. ICD was implanted more frequently to non-FF than FF mutation carriers (7% vs 1%, *p* < 0.001). Common ICD implantation indications were ACA, or LQTS-related syncope during β-blocker medication. An appropriate ICD shock therapy occurred in seven (32%) and an inappropriate shock in three (14%) patients. Six patients suffered a complication in the ICD or pacemaker system (incidence rate 26.8 per 1000 person-years).

### Triggers and predisposing factors for SCD and ACA

The patients with SCD, ACA, or ICD shock therapy are presented in Table 5. Common factors predisposing to SCD were QT-prolonging medication (*n* = 4; citalopram, thioridazine or tizanidine), and absence of β-blocker therapy (*n* = 3). Only one β-blocker medication -compliant patient without predisposing factors suffered a SCD. Of the eight patients who suffered ACA, two were using a QT-prolonging drug (terfenadine or amiodarone), and none were on β-blocker medication at the time of the event.

**Table 5:** SCD, ACA and ICD shock cases

Case	Mutation	Age at event	β-blocker	Trigger or predisposing factor	LQTS dg before event	Device implantation^a^	CE before SCD, ACA or ICD shock^b^
SCD							
1	*KCNQ1* G589D	35.8	No (non-compliance)	Unwitnessed	Yes	No	No
2	*KCNQ1* G589D	35.0	Yes	Unwitnessed, citalopram, ethanol	Yes	No	Syncope (no BB)
3	*KCNQ1* c.1032G > A	32.0	Yes	Physical exertion, citalopram, ethanol	Yes	No	Syncope (no BB)
4	*KCNQ1* G589D	31.3	Yes	Awakening, thioridazine	Yes	No	Syncope (no BB)
5	*KCNH2* Y569H	25.5	No (non-compliance)	Awakening, tizanidine, amphetamine	Yes	No	No
6	*KCNH2* c.842dupG	24.4	Yes	Alarm clock	Yes	No	Syncope (no BB)
7	*KCNH2* A558E	21.8	No	Awakening	No	No	Syncope (no BB)
ACA^c^							
8	*KCNH2* c.643delG	38.8	No	No trigger	No	ICD (39.0)	No
9	*KCNQ1* G589D	34.5	No	Terfenadine, ketoconazole	No	No	Syncope (no BB)
10	*KCNH2* P451L	31.5	No	Excitement	No	PM (31.6)	No
11	*KCNQ1* R518Ter	25.5	No	Rest	No	No	No
12^d^	*KCNH2* L552S	23.6	No	Awakening, amiodarone	No	ICD (23.6)	Syncope (no BB)
13	*KCNH2* c.1558-1G > C	23.5	No	Rest	No	ICD (23.5)	Syncope (no BB)
14	*KCNQ1* G589D	21.0	No	Sport	No	ICD (21.0)	No
15	*KCNQ1* G589D	20.5	No	Post partum period, hypokalemia	No	ICD (20.5)	No
ICD shock							
16	*KCNQ1* D317N	31.1	Yes	Excitement	Yes	ICD (17.2)	Syncope (BB)
17	*KCNQ1* G589D	28.3	Yes	Excitement	Yes	ICD (26.9)	Syncope (no BB)
12^d^	*KCNH2* L552S	25.6	Yes	NA	Yes	ICD (23.6)	ACA (no BB)
18	*KCNH2* W497Ter	24.0	No (non-compliance)	Mirtazapine	Yes	ICD (16.0)	Syncope (BB)
19	*KCNH2* A561V	21.4	Yes	Rest	Yes	ICD (13.1)	ACA (BB),^e^ ICD shock (BB)^e^
20^f^	*KCNH2* L552S *KCNH2* L552S	20.2	No (non-compliance)	Pneumonia, disturbance of diabetes treatment	Yes	ICD (15.6)	Syncope (BB), ICD shock (BB)^e^
21	*KCNH2* L552S	19.9	Yes	Rest	Yes	ICD (18.9)	Syncope (BB)

^a^The age of ICD or pacemaker implantation in parenthesis

^b^Syncope was triggered by swimming, sport, loud noise or startle. “BB” and “no BB” denote patient was and was not, respectively, using β-blocker at the time of the cardiac event

^c^A resuscitation that required external defibrillation

^d^Case 12 suffered both ACA and ICD shock

^e^ACA or ICD shock before the age of 18 years

^f^Homozygous mutation carrier

*CE*: cardiac event, *dg*: diagnosis, *NA*: not available, *PM*: pacemaker, other abbreviations as in Table 1

## Discussion

The present study explored the clinical course of LQTS in 867 adult *KCNQ1* and *KCNH2* mutation carriers and evaluated the risk in six specific mutations. To our knowledge, this is the largest LQTS study of genotyped subjects examining the clinical course in the absence of β-blocker medication, and the first to investigate the clinical course without β-blocker therapy in adult LQTS population.

### Risk factors for cardiac events

Similar to previous studies, female gender, LQT2 genotype, cardiac events before the age of 18, and prolonged QTc duration were found to increase the risk of cardiac events. [7, 8, 10, 11, 17–19, 22] In the present study, mutation carriers with a normal QTc duration (< 440 ms) had a 4.2-fold risk compared to non-carrier relatives, whereas in an earlier study the corresponding risk was 10-fold. [4] A potential reason for the difference is the end point of ACA or SCD in the previous study, as opposed to LQTS-related syncope, ACA, ICD shock, or SCD in our study. An earlier study demonstrated that cardiac events among genotype-negative family members are mostly attributed to nonfatal syncopal episodes. [23] Although mutation carriers with a normal QTc had a higher risk than non-carrier relatives, normal QTc associated with a good prognosis even in patients left untreated with β-blockers: none of the previously asymptomatic non-proband mutation carriers suffered a cardiac event during the prospective follow-up. This is of note as cascade screening of family members reveals a growing number of asymptomatic mutation carriers with a normal or only slightly prolonged QTc.

### Association of the mutation type with clinical events

In the current study, missense *KCNQ1* mutations located in the pore-loop region associated with a higher rate of cardiac events than non-pore-loop mutations, which was not seen in a previous study. [24] However, in the present study most of the pore-loop mutation carriers had the highly malign *KCNQ1* D317N mutation. Previously, *KCNQ1* cytoplasmic loop (c-loop) mutations have been associated with a higher risk. [11] In our study, only six patients were carriers of a c-loop mutation precluding exact comparison of c-loop and non-c-loop mutations. In the present study, *KCNH2* mutations situated in the pore-loop region were not associated with an increased risk as seen in previous studies. [10, 22] On the other hand, only 15 subjects had a pore-loop mutation in our analysis.

All four founder mutations had a significant QT-prolonging effect and associated with increased risk of cardiac events. Similarly to our recent study of pediatric LQT1 and LQT2 population, the *KCNH2* FF mutations led to a milder phenotype than non-FF *KCNH2* mutations. [14] This might be related to the fact that both *KCNH2* FF mutations lead to a functional channel with increased deactivation rate, [21, 25] whereas many non-FF *KCNH2* mutations have more dramatic effects on channel function. However, the risk between *KCNQ1* FF and non-FF mutations did not differ from each other after excluding the *KCNQ1* D317N mutation.

The *KCNQ1* D317N mutation appeared exceptionally malign, and previously it has been shown to associate with diminished chronotropic response and exaggerated QTc prolongation after exercise. [26] This mutation is located in the pore-loop region and leads to complete loss of channel function with a dominant negative effect on the wild type channel protein. [27] On the other hand, the phenotype associated with the *KCNH2* c.453delC mutation turned out to be reasonably mild, in harmony with an earlier study. [28] This N-terminal mutation leads to a premature termination codon, which likely targets the mutated mRNA to non-sense mediated mRNA decay without any dominant negative effect.

### Medical treatment

Only 29% of the patients in the present study used β-blocker medication compared to 45–62% in earlier studies. [10, 11, 19, 22] However, the previous studies included adolescents, who are more frequently treated with β-blockers. [29] In accordance with previous studies, non-compliance to β-blocker therapy increased the risk of cardiac events. [14, 30] Nevertheless, also non-compliant patients demonstrated a decrease in the incidence rate of cardiac events after initiating medication, which suggests a protective role for β-blockers, even when present in suboptimal therapeutic concentrations. However, considerable proportion of the SCD and ACA cases associated with insufficient β-blocker medication underlining the importance of uninterrupted use of prescribed β-blockers.

According to recent ESC guidelines, β-blocker treatment should be initiated if QTc is prolonged, and it may be useful even with normal QTc duration. [3, 4] The results of the current study indicate that asymptomatic adult LQT1 and LQT2 males with FF mutation and QTc duration < 500 ms have a very low risk, and suggest that the avoidance of risk factors may be a sufficient measure. Also, β-blocker medication might not be mandatory for primary prevention in adult *KCNH2* c.453delC mutation carriers and female FF mutation carriers with QTc < 500 ms. For the remaining patients β-blocker treatment is recommended.

In the current study, the use of antidepressant drugs at the end of the follow-up was more common in symptomatic patients. Analysis of a possible causal connection was not feasible due to limited data on the length of the antidepressant medication. However, it is possible that these patients were treated with antidepressants already at the time of the cardiac event. Furthermore, four of the seven SCD cases and two of the eight ACA cases involved treatment with a potentially QT-prolonging drug. Therefore, the present and a previous study [30] emphasize avoidance of QT-prolonging drugs in prevention of potentially life-threatening cardiac events.

### Device therapy

The incidence rate of cardiac events showed reduction after ICD or pacemaker implantation. A potential explanation arises from bradycardia pacing which has been previously demonstrated to reduce the risk of ICD shocks in high-risk patients. [5] However, in the current study, the initiation of β-blocker therapy was coincidental with device implantation in 18 of the 37 cases. Therefore, the reduction in cardiac events might be attributable to β-blocker treatment.

### Study limitations

There are a number of noteworthy limitations in our study. First, only 55% of the patients initially surveyed responded to our inquiry leading to a possibility of selection bias. Second, comparison of patients with different mutation categories occasionally resulted in relatively small patient subgroups. Third, initiation of β-blocker medication was not standardized across participants leading to a concern about confounding by indication. Fourth, we have performed rather many statistical comparisons which may increase the number of false positive findings.

## Conclusions

Molecularly defined LQT1 and LQT2 patients who survive till adulthood continue to be at risk of cardiac events. The clinical risk factors for cardiac events in patients without β-blocker medication were mostly found to be similar to those reported in previous studies that included patients treated with β-blockers. Specific *KCNQ1* and *KCNH2* mutations were associated with varied risk of cardiac events, independently of gender, QTc duration, and cardiac events before the age of 18. The identification of high-risk and low-risk mutations may enhance risk stratification, and may help to reveal patient groups in which lifestyle modifications are a sufficient measure.

## Additional files

Additional file 1: Questionnaire. (DOCX 20 kb)

Additional file 2: **Table S1.** Mutations in the study. **Table S2.** Characteristics of the patients with > 1 mutation. **Table S3.** Characteristics of the non-carrier relatives. **Table S4.** ICDs, pacemakers and LCSDs. (DOC 233 kb)

## Abbreviations

ACA: Aborted cardiac arrest
BB: β-blocker
FF: Finnish founder
HR: Hazard ratio
ICD: Implantable cardioverter-defibrillator
LCSD: Left cardiac sympathetic denervation
LQT1: Long QT syndrome type 1
LQT2: Long QT syndrome type 2
LQTS: Long QT syndrome
SCD: Sudden cardiac death
