# Supplementary material for: Clinical and molecular genetic risk determinants in adult long QT syndrome type 1 and 2 patients: Koponen et al. Follow-up of adult LQTS patients
Source: BMC Med Genet. 2018 Apr 5;19:56. doi: 10.1186/s12881-018-0574-0 (PMC5887247; doi:10.1186/s12881-018-0574-0)
Supplement: Supplementary file 1 — Questionnaire. (DOCX 20 kb) [file 12881_2018_574_MOESM1_ESM.docx]

1. Phone number: ___________________________________

2. Occupation: _____________________________________

3. Weight: _______________ kg

4. Height: _______________cm

5. Email address: ___________________________________

6. What medication do you use daily? Name of the drugs, dose (milligram) and number of doses:

**__________________________________________________________________________________________________________**

**__________________________________________________________________________________________________________**

**__________________________________________________________________________________________________________**

__________________________________________________________________________________________________________

__________________________________________________________________________________________________________

7. Tick here if you are **not** using any medication daily **( )**

**Antidepressive, anxiolytic and antipsychotic medication**

8. Have you ever used antidepressive, anxiolytic or antipsychotic medication? **No ( ) Yes ( )**

9. Are you currently using antidepressive, anxiolytic or antipsychotic medication? **No ( ) Yes ( )**

**Beta blocker medication**: Beta blockers are commonly used in the prevention of cardiac arrhythmia. Common beta blockers include for example Emconcor, Orloc, Bisoprolol, Seloken, Metoprolol, Metohexal, Spesicor, Kerlon, Atenol, Tenoblock, Atenblock, Tenoprin, Selectol, Propral, Dociton.

10. Have you ever been prescribed a beta blocker for a daily use? **No ( ) Yes ( )**

11. When did you start beta blocker medication? **Year**: _________________

2. Are you currently using beta blocker medication daily for LQTS or any other cause? **No ( ) Yes ( )**

13. In case you have discontinued beta blocker medication, in which year the medication discontinued? **Year**: _________________

14. Have you ever had a syncope during beta blocker medication? **No ( ) Yes ( )**

15. How many syncopes have you had during beta blocker medication? **Number:** ________________

16. Do you forget taking beta blocker more often than once a month? **No ( ) Yes ( )**

17. Do you have side effects from beta blocker medication? **No ( ) Yes ( )**

18. Do you sometimes skip beta blocker medication because of the side effects? **No ( ) Yes ( )**

19. Do you skip beta blocker medication because of the side effects more often than once a month? **No ( ) Yes ( )**

20. In case you have side effects from beta blocker medication what side effect do you have? ______________________________

_________________________________________________________________________________________________________

**Symptoms**

21. Have you ever had a syncope? **No ( ) Yes ( )**

22. How many times have you had a syncope during your life? **Number:** **________________**

23. At what age did you have your first syncope? **Age:** _________________

24. At what age did you have your last syncope? **Age:** _________________

25. Did syncope happen in some of the following circumstances:

There can be more than one “Yes” answer.

|  | **No** | **Yes** |  | **No** | **Yes** |
| --- | --- | --- | --- | --- | --- |
| **Swimming** |  |  | Standing up or standing long time |  |  |
| **Exercise** |  |  | Sauna or hot day |  |  |
| **Sudden loud noise or startle** |  |  | **Vaccination, blood sample or other operation** |  |  |
| **Sleep or rest** |  |  |  |  |  |
| **Were you using medication during syncope?**  **Which medication?** | | | | | |

**TURN OVER, PLEASE**

**Resuscitations**

26. Have you ever been resuscitated? By resuscitation we mean a circumstance in which patient is given cardiopulmonary resuscitation or an electric shock (defibrillation) to restart the heart. **No ( ) Yes ( )**

27. How many times have you been resuscitated? **Number**: _______________________

28. What year were you resuscitated? **Year**: _______________________

29. Did the resuscitation happen in hospital or outside of hospital? **In hospital ( ) Outside of hospital ( )**

**Pacemaker treatment**

30. Do you have a cardiac pacemaker? **No ( ) Yes ( )**

- In which hospital the pacemaker was planted? **Hospital**: ________**______________________________________**

31. In case you have a pacemaker is it a defibrillating pacemaker (ICD)? **No ( ) Yes ( )**

32. In case you have an ICD has the pacemaker ever given a defibrillation treatment? **No ( ) Yes ( )**

33. In which hospital your pacemaker is being followed-up? **Hospital:** ______________________________________________

**Surgeries and anesthesia**

34. Have you ever had a surgical operation or other procedure in general anesthesia? **No ( ) Yes ( )**

35. Were there any problems related to the anesthesia? **No ( ) Yes ( )**

36. In case there were problems what kind of problems there were? **Problems:** _____________________________________________

37. In which hospital the general anesthesia took place? **Hospital:** _____________________________________________

**Exercise**

38. What sports or exercise do you have as a hobby? **Sport:** _____________________________________________

39. What do you think of your physical condition?

( ) It is better than in other people of my age

( ) It is same as in other people of my age

( ) It is worse than in other people of my age

40. Are you trained by a coach in a sports club? **No ( ) Yes ( )**

41. Do you compete in any sport? **No ( ) Yes ( )**

42. In which sports do you compete? **Sports:** _____________________________________________

43. How often do you swim?

( ) Never

( ) Once a month or less often

( ) Weekly year-round

( ) Almost daily year-round

( ) Weekly in summer

( ) Almost daily in summer

Date and signature ____________________________________________________________
