# Supplementary material for: Clinical and molecular genetic risk determinants in adult long QT syndrome type 1 and 2 patients: Koponen et al. Follow-up of adult LQTS patients
Source: BMC Med Genet. 2018 Apr 5;19:56. doi: 10.1186/s12881-018-0574-0 (PMC5887247; doi:10.1186/s12881-018-0574-0)
Supplement: Supplementary file 2 — Table S1. Mutations in the study. Table S2. Characteristics of the patients with > 1 mutation. Table S3. Characteristics of the non-carrier relatives. Table S4. ICDs, pacemakers and LCSDs. (DOC 233 kb) [file 12881_2018_574_MOESM2_ESM.doc]

**ADDITIONAL MATERIAL**

Supplemental Tables

**Table 1S. Missense and non-missense mutations in LQT1 and LQT2 patients.***

| Locus/Mutation | n (%) | Families, n | Mutation type | Mutation location |
| --- | --- | --- | --- | --- |
| FF-mutations |  |  |  |  |
| *KCNQ1* |  |  |  |  |
| p.G589D | 453 (74) | 111 | missense | C-terminus |
| c.1129-2A>G | 72 (12) | 20 | splice site | C-terminus |
| *KCNH2* |  |  |  |  |
| p.R176W | 86 (36) | 30 | missense | N-terminus |
| p.L552S | 73 (31) | 27 | missense | MS |
|  |  |  |  |  |
| Non-FF mutations |  |  |  |  |
| *KCNQ1* |  |  |  |  |
| p.T169K | 1 (0.2) | 1 | missense | MS |
| p.D202H | 2 (0.3) | 1 | missense | MS |
| p.W248C | 6 (1) | 1 | missense | c-loop |
| p.G269S | 6 (1) | 1 | missense | MS |
| p.S277L | 6 (1) | 1 | missense | MS |
| p.T311I | 3 (0.5) | 1 | missense | pore-loop |
| p.Y315C | 1 (0.2) | 1 | missense | pore-loop |
| p.D317N | 20 (3) | 1 | missense | pore-loop |
| p.G325R | 1 (0.2) | 1 | missense | pore-loop |
| p.A341V | 2 (0.3) | 1 | missense | MS |
| p.R366W | 10 (2) | 3 | missense | C-terminus |
| p.A525T | 1 (0.2) | 1 | missense | C-terminus |
| p.S546L | 4 (0.6) | 2 | missense | C-terminus |
| p.R561G | 5 (0.8) | 1 | missense | C-terminus |
| p.R594Q | 1 (0.2) | 1 | missense | C-terminus |
| p.Y171Ter | 1 (0.2) | 1 | nonsense | c-loop |
| c.683+5G>A | 1 (0.2) | 1 | splice site | MS |
| p.S277del | 5 (0.8) | 2 | deletion | MS |
| c.1032G>A | 3 (0.5) | 1 | splice site | MS |
| p.R518Ter | 10 (2) | 4 | nonsense | C-terminus |
| c.1684_1685+1delAGG | 3 (0.5) | 1 | frameshift/  splice site | C-terminus |
| *KCNH2* |  |  |  |  |
| p.A193V | 1 (0.4) | 1 | missense | N-terminus |
| p.H402R | 1 (0.4) | 1 | missense | N-terminus |
| p.P451L | 2 (0.9) | 1 | missense | MS |
| p.A558E | 3 (1) | 1 | missense | MS |
| p.A561T | 1 (0.4) | 1 | missense | MS |
| p.A561V | 2 (0.9) | 1 | missense | MS |
| p.Y569H | 1 (0.4) | 1 | missense | MS |
| p.G572S | 2 (0.9) | 1 | missense | pore-loop |
| p.G584S | 10 (4) | 1 | missense | pore-loop |
| p.G601S | 2 (0.9) | 1 | missense | pore-loop |
| p.T613M | 1 (0.4) | 1 | missense | pore-loop |
| p.N629S | 1 (0.4) | 1 | missense | pore-loop |
| p.A913V | 1 (0.4) | 1 | missense | C-terminus |
| p.G1036D | 1 (0.4) | 1 | missense | C-terminus |
| p.C39Ter | 3 (1) | 1 | nonsense | N-terminus |
| c.221_242del | 1 (0.4) | 1 | frameshift | N-terminus |
| c.453delC | 24 (10) | 2 | frameshift | N-terminus |
| c.643delG | 1 (0.4) | 1 | frameshift | N-terminus |
| p.R273Ter | 4 (2) | 2 | nonsense | N-terminus |
| c.842dupG | 2 (0.9) | 2 | frameshift | N-terminus |
| c.853_859dupGCCGACG | 3 (1) | 1 | frameshift | N-terminus |
| c.1129-58_1320del | 1 (0.4) | 1 | deletion/  splice site | MS |
| c.1379delA | 1 (0.4) | 1 | frameshift | MS |
| p.W497Ter | 1 (0.4) | 1 | nonsense | MS |
| c.1558-1G>C | 1 (0.4) | 1 | splice site | MS |
| c.1631_1632delAG | 4 (2) | 2 | frameshift | MS |
| c.2173_2196del | 1 (0.4) | 1 | deletion | C-terminus |
| c.2959_2960delCT | 1 (0.4) | 1 | frameshift | C-terminus |
| p.W1001Ter | 1 (0.4) | 1 | nonsense | C-terminus |
| c.3017delG | 1 (0.4) | 1 | frameshift | C-terminus |
| c.3093_3106del | 1 (0.4) | 1 | frameshift | C-terminus |
| c.3152+1G>A | 3 (1) | 1 | splice site | C-terminus |
| c.3160dupA | 1 (0.4) | 1 | frameshift | C-terminus |

Reference sequences: NM_000218.2 (*KCNQ1*) and NM_000238.3 (*KCNH2*).

*Patients with >1 LQTS-causing mutation (n=7) are excluded.

c-loop=cytoplasmic loop, FF=Finnish founder, MS=membrane spanning.

**Table 2S. Characteristics of the patients with >1 mutation in the *KCNQ1* or *KCNH2* genes.**

| Case | Mutations | Gender | QTc (ms) | Proband | Cardiac event at age 0-18* | Cardiac event at age 18-40* | β-blocker before age 40 | Device implantation or LCSD before age 40† |
| --- | --- | --- | --- | --- | --- | --- | --- | --- |
| 1 | *KCNQ1* G589D *KCNH2* R176W | female | 502 | no | no | syncope (30.5) | yes | ICD (30.6) |
| 2 | *KCNQ1* G589D *KCNH2* R176W | female | 507 | yes | syncope (14.4) | syncope (33.4) | no (diagnosis at age 43.6) | no |
| 3 | *KCNQ1* G589D *KCNH2* L552S | male | 550 | yes | no | no | no (diagnosis at age 66.5) | no |
| 4‡ | *KCNQ1* G589D *KCNQ1* Y171Ter | male | 566 | yes | syncope (9.6) | no | yes (3.6) | PM (10.8), LCSD (11.7) |
| 5‡ | *KCNQ1* G589D *KCNQ1* G589D | male | 592 | yes | syncope (5.7) | no | yes (0.5) | LCSD (5.8) |
| 6 | *KCNH2* L552S  *KCNH2* L552S | female | 513 | yes | syncope (1.5) | ICD shock (20.2, 20.5) | yes (7.0) | ICD (16.5) |
| 7 | *KCNQ1* c.1129-2A>G  *KCNH2* R176W | female | 486 | no | no | syncope (35.4) | yes (35.6) | PM (35.6) |

*The trigger for syncope was swimming, sport, loud noise or startle. The age of cardiac event in parenthesis.

†The age of ICD or PM implantation, or LCSD in the parenthesis.

‡Cases 4 and 5: congenital deafness.

ACA=aborted cardiac arrest, ICD=implantable cardioverter-defibrillator, LCSD=left cardiac sympathetic denervation, ms=milliseconds, PM=pacemaker, SCD=sudden cardiac death.

**Table 3S. Characteristics of the non-carrier relatives by gender at the age of 18-40 years**

|  | **Female** | **Male** | **P-value** |
| --- | --- | --- | --- |
| N (%) | 412 (63) | 242 (37) |  |
| Age, y | 37.2±5.5 | 37.0±5.8 | 0.62 |
| QTc, ms | 424±26 | 410±22 | <0.001 |
| β-blocker | 30 (7) | 3 (1) | 0.001 |
| ICD, Pacemaker or LCSD | 0 | 0 | 1.00 |
| CE | 7 (2) | 4 (2) | 1.00 |
| Syncope ***** | 7 (2) | 4 (2) | 1.00 |
| ACA or SCD † | 0 | 0 | 1.00 |
| CE age, y ‡ | 26.6±8.8 | 26.9±5.6 | 0.85 |
| Death | 0 | 4 (2) | 0.02 |
| CE before age 18 | 9 (2) | 3 (1) | 0.55 |

Parameters shown as n (%), or mean±SD.

*****Triggered by swimming, sport, loud noise or startle.

†A resuscitation that required external defibrillation or appropriate ICD shock, or death not explained by any other cause and abrupt in onset if witnessed.

‡The first cardiac event at the age of 18-40 years.

ACA=aborted cardiac arrest, BB=β-blocker, CE=cardiac event, ICD=implantable cardioverter-defibrillator, LCSD=left cardiac sympathetic denervation, SCD=sudden cardiac death, SD=standard deviation.

**Table 4S. ICDs, pacemakers and LCSDs in adult LQT1 and LQT2 patients.**

| Case | Mutations | Gender | QTc | Device* | Indication† | CE after implantation‡ | Appropriate shock*‡ | Inappropriate shock*‡ | Shock trigger | BB during shock | Complication‡ | Revision‡ | Follow-up (y)‡ |
| --- | --- | --- | --- | --- | --- | --- | --- | --- | --- | --- | --- | --- | --- |
| **ICD** |  |  |  |  |  |  |  |  |  |  |  |  |  |
| 1 | *KCNH2* L552S | female | 618 | ICD (18.9) | TdP, syncope (BB) | ICD shocks | yes (19.9, 26.1, 27.5) | no | rest | yes | no | generator x1 | 15.5 |
| 2 | *KCNQ1* D317N | female | 572 | ICD (17.2) | Syncope (BB) | ICD shock | yes (31.1) | no | excitement | yes | no | generator x2 | 13.5 |
| 3 | *KCNH2* c.3152+1G>A | female | 513 | ICD (16.5) | ACA (no BB) | no | no | no | - | - | no | generator x1 | 10.1 |
| 4 | *KCNH2* A561V | female | 517 | ICD (13.1) | ACA (BB) | ICD shocks | yes (21.4, 23.2, 23.5) | yes (25.6) | startle, rest | yes | generator malfunction | generator x2, lead x1 | 8.6 |
| 5 | *KCNQ1* G589D | female | 512 | ICD (31.4) | Syncope (BB) | no | no | no | - | - | no | generator x1 | 8.6 |
| 6 | *KCNH2* W1001Ter | female | 501 | ICD (25.7) | Syncopes (no BB) | no | no | yes (29.4) | lead damage | yes | lead damage | generator x1, lead x1 | 7.8 |
| 7§ | *KCNH2* L552S  *KCNH2* L552S | female | 513 | ICD (15.6) | Syncope (BB) | ICD shocks | yes (20.2, 20.5) | no | pneumonia, disturbance of diabetes treatment | no (non-compliance) | lead damage | generator x2, lead x1 | 7.5 |
| 8 | *KCNH2* A558E | male | 593 | ICD (30.3) | Syncope (no BB), bradycardia, QTc | no | no | no | - | - | no | generator x1 | 6.8 |
| 9 | *KCNQ1* G589D | female | 451 | ICD (25.9) | Syncope (BB) | no | no | no | - | - | no | no | 6.7 |
| 10§ | *KCNQ1* G589D  *KCNH2* R176W | female | 502 | ICD (30.6) | Syncope (BB) | no | no | no | - | - | no | no | 6.4 |
| 11 | *KCNH2* W497Ter | female | 493 | ICD (16.0) | TdP (BB) | ICD shock | yes (24.0) | no | mirtazapine | no (non-compliance) | no | no | 6.3 |
| 12 | *KCNQ1* A341V | male | 543 | ICD (12.4) | ACA (BB) | no | no | no | - | - | no | no | 5.3 |
| 13 | *KCNQ1* G589D | female | 404 | ICD (26.9) | Syncope (no BB) | ICD shock | yes (28.3) | no | excitement | yes | no | no | 5.0 |
| 14 | *KCNH2* L552S | female | 582 | ICD (23.6) | ACA (no BB) | ICD shock | yes (25.6) | no | NA | yes | no | no | 5.0 |
| 15 | *KCNH2* c.3093_3106del | male | 496 | ICD (37.9) | Syncope (no BB), QTc | no | no | no | - | - | no | no | 2.1 |
| 16 | *KCNH2* A558E | male | 485 | ICD (28.6) | Family history | no | no | no | - | - | no | no | 1.7 |
| 17 | *KCNQ1* A341V | male | 479 | ICD (8.7) | Syncope (BB) | no | no | no | - | - | no | no | 1.5 |
| 18 | *KCNH2* c.643delG | male | 482 | ICD (39.0) | ACA (no BB) | no | no | no | - | - | no | no | 1.0 |
| 19 | *KCNQ1* G589D | female | 462 | ICD (20.5) | ACA (no BB) | no | no | no | - | - | ICD infection | generator x1, lead x1 | 0.9 |
| 20 | *KCNH2* c.1558-1G>C | female | 447 | ICD (23.5) | ACA (no BB) | no | no | yes (23.7) | lead displacement | yes | lead displacement | lead reimplantation | 0.2 |
| 21 | *KCNQ1* G589D | male | 654 | ICD (21.0) | ACA (no BB) | no | no | no | - | - | no | no | 0.1 |
| 22 | *KCNQ1* R366W | female | 450 | LCSD (7.1), ICD (12.5) | Syncope (BB), inadequate BB compliance | no | no | no | - | - | no | no | 0.1 |
| **PM** |  |  |  |  |  |  |  |  |  |  |  |  |  |
| 23 | *KCNH2* R176W | female | 515 | PM (24.5) | Syncope (BB), badycardia | no | - | - | - | - | PM infection | generator x2, lead x1 | 15.5 |
| 24 | *KCNQ1* c.1129-2A>G | female | 503 | PM (28.0) | Syncope (no BB), bradycardia | no | - | - | - | - | no | generator x1 | 12.0 |
| 25 | *KCNH2* c.842dupG | female | 475 | PM (28.1) | LQTS, syncope (no BB) | no | - | - | - | - | no | no | 11.9 |
| 26 | *KCNH2* H402R | female | 498 | PM (30.0) | Syncope (no BB), bradycardia | no | - | - | - | - | no | generator x1 | 10.0 |
| 27 | *KCNH2* R176W | female | 460 | PM (30.0) | Syncopes (no BB) | no | - | - | - | - | no | generator x1 | 9.6 |
| 28§ ¶ | *KCNQ* G589D  *KCNQ1* Y171Ter | male | 566 | PM (10.8), LCSD (11.7) | Syncope (BB), J-L-N | no | - | - | - | - | Horner sdr | generator x3, lead x1 | 9.5 |
| 29 | *KCNH2* A561V | female | 526 | PM (30.9) | Syncope (no BB), QTc | no | - | - | - | - | no | no | 9.1 |
| 30 | *KCNH2* P451L | female | 439 | PM (31.6) | Syncope (no BB), bradycardia | no | - | - | - | - | no | generator x1, lead x1 | 8.4 |
| 31§ | *KCNQ1* c.1129-2A>G  *KCNH2* R176W | female | 486 | PM (35.6) | Syncope (no BB), bradycardia | no | - | - | - | - | no | no | 4.4 |
| 32 | *KCNQ1* G589D | female | 443 | PM (35.1) | Sinus arrest | no | - | - | - | - | no | no | 4.2 |
| 33 | *KCNQ1* G589D | female | 456 | PM (35.9) | Sinus arrest | no | - | - | - | - | no | no | 4.1 |
| 34 | *KCNH2* c.1129-58_1320del | female | 606 | PM (24.2) | Syncope (BB), bradycardia | no | - | - | - | - | no | no | 1.4 |
| 35 | *KCNH2* T613M | female | 536 | PM (38.7) | Syncope (no BB), QTc | no | - | - | - | - | no | no | 1.3 |
| 36 | *KCNQ1* G589D | female | 423 | PM (38.7) | Family history, II-III^o^ AV-block | no | - | - | - | - | no | no | 1.3 |
| 37 | *KCNQ1* 1129-2G>A | male | 420 | PM (39.4) | Cardioinhibitory vasovagal syncope | no | - | - | - | - | no | no | 0.6 |
| **LCSD** |  |  |  |  |  |  |  |  |  |  |  |  |  |
| 38 | *KCNH2* C39Ter | female | 519 | LCSD (19.0) | Syncope (no BB), QTc | Syncope | - | - | - | - | no | no | 21.0 |
| 39§ ¶ | *KCNQ1* G589D  *KCNQ1* G589D | male | 592 | LCSD (5.8) | Syncope (BB), J-L-N | no | - | - | - | - | no | no | 11.9 |

*The age of ICD or PM implantation, LCSD, or ICD shock in parenthesis.

†”BB” and “no BB” denote patient was and was not, respectively, using β-blocker at the time of the cardiac event.

‡At the age of 18-40 years.

§Carrier of a homozygous or compound heterozygous mutation.

¶Cases 28 and 39: congenital deafness.

BB=β-blocker, J-L-N=Jervell-Lange-Nielsen syndrome, NA=not available, sdr=syndrome, TdP=Torsades de pointes ventricular tachycardia. Other abbreviations as in Table 2S.

NA=not available, J-L-N=Jervell-Lange-Nielsen syndrome, sdr=syndrome, TdP=Torsades de pointes ventricular tachycardia
